# Vitamin D supplementation: a potential therapeutic agent for metastatic colorectal cancer

**DOI:** 10.1038/s41416-020-0958-8

**Published:** 2020-07-06

**Authors:** Chen Yuan, Kimmie Ng

**Affiliations:** Department of Medical Oncology, Dana-Farber Cancer Institute and Harvard Medical School, Boston, MA USA

**Keywords:** Colorectal cancer, Translational research

## Abstract

Preclinical and epidemiological evidence suggests that vitamin D may have anti-cancer activities in patients with colorectal cancer. A recently completed, randomised Phase 2 trial of vitamin D_3_ supplementation in patients with metastatic colorectal cancer has shown promising results, and a Phase 3 trial is currently underway.

## Main

Colorectal cancer (CRC) is the third most common cancer in both men and women in the United States.^1^ It is also the second leading cause of cancer death in the United States,^1^ as well as worldwide.^2^ According to the Surveillance, Epidemiology, and End Results (SEER) Program of the National Cancer Institute, ~21% of CRC patients have metastatic disease with a 5-year survival rate of only 14%.^1^ Therefore, novel treatment approaches that are safe, can delay progression, and prolong survival are urgently needed for this group of patients.

Vitamin D is an inexpensive, non-toxic, and easily accessible treatment that has demonstrated anti-neoplastic activities against CRC. Colon cancer cells express high levels of the vitamin D receptor (VDR).^3^ In vitro studies found that by binding with VDR, vitamin D can induce apoptosis of colon cancer cells and counteract aberrant WNT-β catenin signalling.^4^ In *APC*(min) mice, a model of intestinal tumorigenesis, tumour burden was increased by inactivation of the *VDR* gene and decreased by treatment with vitamin D or its synthetic analogue.^4^

To further explore these findings in CRC patients, observational studies were conducted to evaluate the association between plasma levels of 25-hydroxyvitamin D [25(OH)D] and patient outcome. Prospective cohort studies, with circulating 25(OH)D levels measured either prior to or at cancer diagnosis, consistently showed a high prevalence of vitamin D deficiency among CRC patients.^5^ Moreover, CRC patients with higher 25(OH)D levels had longer survival times than those with lower levels.^5^ In the initial report of 304 CRC patients from the Nurses’ Health Study and the Health Professionals Follow-Up Study, subgroup analyses suggested a greater association of 25(OH)D levels with overall survival among patients with stage III or IV disease versus stage I or II.^6^ However, in such studies that included patients with all stages of CRC, those with metastatic disease accounted for only a small fraction, resulting in inadequate power to evaluate the relationship between 25(OH)D levels and survival for this group of patients.

To date, two large prospective cohort studies have been conducted to specifically evaluate the association of circulating 25(OH)D levels with survival of patients with metastatic CRC.^7,8^ One was conducted among 515 patients with stage IV CRC enrolled in the North Central Cancer Treatment Group 9741 trial, which found a significant association between higher 25(OH)D levels at diagnosis and improved overall survival among patients receiving FOLFOX (infusional fluorouracil, leucovorin, and oxaliplatin).^8^ A later analysis of 1043 patients with metastatic CRC who were enrolled on Cancer and Leukemia Group B (Alliance)/SWOG 80405, a randomised Phase 3 trial of chemotherapy plus biologicals for first-line treatment,^9^ confirmed the association between higher plasma 25(OH)D levels and improved progression-free and overall survival.^7^

Although observational studies support a positive association between 25(OH)D levels and survival of patients with metastatic CRC, they are not able to establish a causal role of higher 25(OH)D levels in improving survival. Vitamin D insufficiency can be a surrogate of worse health or a reflection of less favourable disease, which may confound the true relationship between vitamin D and survival. A double-blind Phase 2 randomised clinical trial, SUNSHINE, was therefore conducted to examine whether addition of high-dose vitamin D_3_ (4000 IU/day), versus standard-dose vitamin D_3_ (400 IU/day), to standard chemotherapy can improve outcomes in patients with metastatic CRC.^10^ At study baseline, 91% of these patients had insufficient levels (<30 ng/ml) of 25(OH)D, with a median level of 16.1 ng/ml in the high-dose vitamin D_3_ group and 18.7 ng/ml in the standard-dose vitamin D_3_ group. During supplementation, median plasma 25(OH)D levels increased into the sufficient range (≥30 ng/ml) among patients receiving high-dose vitamin D_3_ (median: 35.2 ng/ml), but remained unchanged with standard-dose vitamin D_3_ (median: 18.5 ng/ml), supporting the ability of high-dose vitamin D_3_ supplementation to raise 25(OH)D levels in patients with metastatic CRC undergoing chemotherapy. Moreover, patients receiving high-dose vitamin D_3_ had improved progression-free survival compared with those receiving standard-dose vitamin D_3_ (median progression-free survival: 13.0 months versus 11.0 months; stratified log-rank *P* = 0.03), Importantly, high-dose vitamin D_3_ supplementation did not result in any added toxicity.

To determine whether vitamin D supplementation should be considered a part of standard treatment of metastatic CRC, a confirmatory multicentre, double-blind, randomised Phase 3 trial of vitamin D_3_ supplementation in combination with standard chemotherapy among previously untreated patients with metastatic CRC called SOLARIS is currently underway through the Alliance for Clinical Trials in Oncology (ClinicalTrials.gov identifier: NCT04094688). The trial will enrol 400 patients with a greater diversity in race and geographical residence, as well as adequate power to detect a progression-free and overall survival benefit.

The evolution and development of vitamin D into a potential therapeutic agent for cancer is a prime example of the importance of research on diet and lifestyle factors and the critical role that they may play in cancer pathogenesis and treatment. Repurposing a safe, affordable, and accessible agent such as vitamin D could potentially have a large and wide-reaching impact globally, even in regions with fewer resources. Robust funding and support are therefore needed to conduct further studies on the biological mechanisms underlying the activity of vitamin D and other modifiable factors in CRC, such that their acceptance and incorporation into standard paradigms of patient care and management may be facilitated.

## Data Availability

Not applicable.

## References

[1] Siegel RL, Miller KD, Jemal A (2020). Cancer statistics, 2020. CA Cancer J. Clin..

[2] Bray F, Ferlay J, Soerjomataram I, Siegel RL, Torre LA, Jemal A (2018). Global cancer statistics 2018: GLOBOCAN estimates of incidence and mortality worldwide for 36 cancers in 185 countries. CA Cancer J. Clin..

[3] Meggouh F, Lointier P, Saez S (1991). Sex steroid and 1,25-dihydroxyvitamin D_3_ receptors in human colorectal adenocarcinoma and normal mucosa. Cancer Res..

[4] Dou R, Ng K, Giovannucci EL, Manson JE, Qian ZR, Ogino S (2016). Vitamin D and colorectal cancer: molecular, epidemiological and clinical evidence. Br. J. Nutr..

[5] Maalmi H, Walter V, Jansen L, Boakye D, Schottker B, Hoffmeister M (2018). Association between blood 25-hydroxyvitamin D levels and survival in colorectal cancer patients: an updated systematic review and meta-analysis. Nutrients.

[6] Ng K, Meyerhardt JA, Wu K, Feskanich D, Hollis BW, Giovannucci EL (2008). Circulating 25-hydroxyvitamin d levels and survival in patients with colorectal cancer. J. Clin. Oncol..

[7] Yuan C, Sato K, Hollis BW, Zhang S, Niedzwiecki D, Ou FS (2019). Plasma 25-hydroxyvitamin D levels and survival in patients with advanced or metastatic colorectal cancer: findings from CALGB/SWOG 80405 (Alliance). Clin. Cancer Res..

[8] Ng K, Sargent DJ, Goldberg RM, Meyerhardt JA, Green EM, Pitot HC (2011). Vitamin D status in patients with stage IV colorectal cancer: findings from Intergroup trial N9741. J. Clin. Oncol..

[9] Venook AP, Niedzwiecki D, Lenz HJ, Innocenti F, Fruth B, Meyerhardt JA (2017). Effect of first-line chemotherapy combined with cetuximab or bevacizumab on overall survival in patients with KRAS wild-type advanced or metastatic colorectal cancer: a randomized clinical trial. JAMA.

[10] Ng K, Nimeiri HS, McCleary NJ, Abrams TA, Yurgelun MB, Cleary JM (2019). Effect of high-dose vs standard-dose vitamin D_3_ supplementation on progression-free survival among patients with advanced or metastatic colorectal cancer: The SUNSHINE Randomized Clinical Trial. JAMA.

